# Gas-Phase Thermal Tautomerization of Imidazole-Acetic Acid: Theoretical and Computational Investigations

**DOI:** 10.3390/ijms161125959

**Published:** 2015-11-04

**Authors:** Saadullah G. Aziz, Osman I. Osman, Shaaban A. Elroby, Rifaat H. Hilal

**Affiliations:** 1 Chemistry Department, Faculty of Science, King Abdulaziz University, P.O. Box 80203, Jeddah 21589, Saudi Arabia; saziz@kau.edu.sa (S.G.A.); skamel@kau.edu.sa (S.A.E.); rhilal@kau.edu.sa (R.H.H.); 2 Chemistry Department, Faculty of Science, University of Khartoum, P.O. Box 321, Khartoum 11111, Sudan; 3 Chemistry Department, Faculty of Science, Beni-Suef University, Beni-Suef 62511, Egypt; 4 Chemistry Department, Faculty of Science, Cairo University, Giza 12613, Egypt

**Keywords:** imidazole-4-acetic acid, tautomerization, long-range, dispersion

## Abstract

The gas-phase thermal tautomerization reaction between imidazole-4-acetic (I) and imidazole-5-acetic (II) acids was monitored using the traditional hybrid functional (B3LYP) and the long-range corrected functionals (CAM-B3LYP and ωB97XD) with 6-311++G** and aug-cc-pvdz basis sets. The roles of the long-range and dispersion corrections on their geometrical parameters, thermodynamic functions, kinetics, dipole moments, Highest Occupied Molecular Orbital–Lowest Unoccupied Molecular Orbital (HOMO–LUMO) energy gaps and total hyperpolarizability were investigated. All tested levels of theory predicted the preference of I over II by 0.750–0.877 kcal/mol. The origin of predilection of I is assigned to the H-bonding interaction (n_N8_→σ*_O14–H15_). This interaction stabilized I by 15.07 kcal/mol. The gas-phase interconversion between the two tautomers assumed a 1,2-proton shift mechanism, with two transition states (TS), TS1 and TS2, having energy barriers of 47.67–49.92 and 49.55–52.69 kcal/mol, respectively, and an sp^3^-type intermediate. A water-assisted 1,3-proton shift route brought the barrier height down to less than 20 kcal/mol in gas-phase and less than 12 kcal/mol in solution. The relatively high values of total hyperpolarizability of I compared to II were interpreted and discussed.

## 1. Introduction

Imidazole is a heterocyclic polar organic compound. Its 5-membered ring is planar. It has two tautomers because the proton can be linked with any of the two nitrogen atoms. Imidazole exists in many biologically important compounds like histidine and histamine [1]. Imidazoleacetic acid is one of the most important derivatives of imidazole. It is one possible paths of histamine metabolism by being an intermediate in the physiological oxidation of histamine [2]. It has two well-known isomers: imidazole-4-acetic acid and imidazole-5-acetic acid, which are isolated in the laboratory as hydrochlorides or sodium salts [3]. The former occurs naturally in the brain as a metabolite of histamine oxidation [4]. It was prepared, in the laboratory, from the acid hydrolysis of cyanomethylimidazole, which is made from chloromethylimidazole [5]. Its neuropharmacological functions include enhancing the binding of benzodiazepine to γ-aminobutyric acid (GABA, its structural analogue) receptor complex in membrane preparations and acting as an antagonist through its affinity for GABA receptors [6]. On the other hand, imidazole-5-acetic acid derivatives have anti-inflammatory activities [7], an antagonistic effect on the peptide hormone, angiotensin II, in addition to some hypotensive activities. They are also useful as hypotensive agents [8].

Another very important application of imidazole-4-acetate anion is its affinity for transition metal cations forming complexes via bonding through the carboxylic oxygen and the un-protonated imidazoline nitrogen atoms [9,10]. Imidazole-5-acetate anions are hindered from binding with the transition metal cations as the position of the donor oxygen and nitrogen atoms do not facilitate chelation [11].

These vital biological, pharmacological, and physicochemical applications of imidazoleacetic acid have caught the attention of many researchers [9,10,11]. Our contribution here is to shed more light on the geometric structures of its conformers, the interconversion of its tautomers and their kinetic and thermodynamic stabilities. Their non-linear optical (NLO) properties were also investigated and supported by natural bond orbital (NBO) analysis.

## 2. Results and Discussion

### 2.1. Molecular Structure

**Figure 1:**
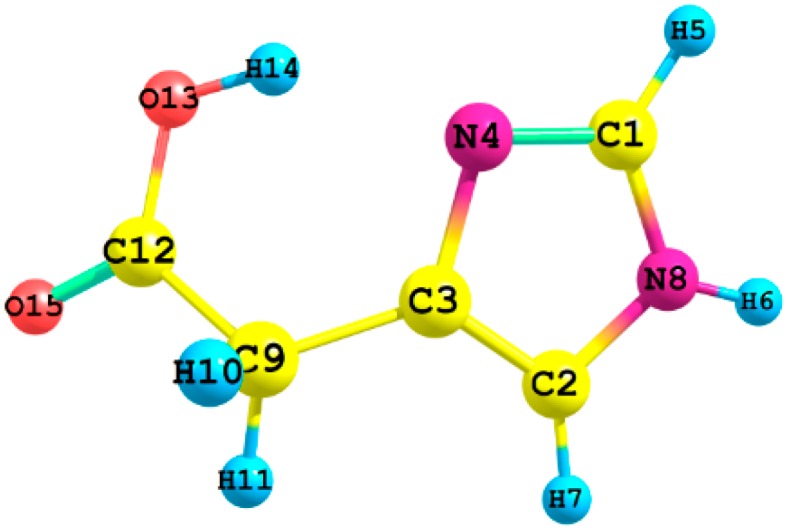
The atom numbering of imidazole-4-acetic acid.

**Figure 2:**
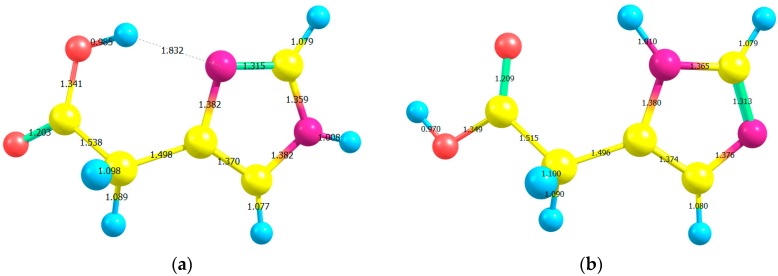
The optimized bond lengths of (**a**) H-bonded imidazole-4-acetic acid (I) and (**b**) imidazole-5-acetic acid (II) using B3LYP/6-311++G** level of theory.

Figure 1 shows the atom numbering for the different conformers of imidazoleacetic acid, I and II (See their standard coordinates in Tables S1 and S2). Table 1 and Table 2 list the gas-phase optimized bond lengths (see Figure 2), and bond angles of I and II which have been estimated by using B3LYP, CAM-B3LYP, and ωB97XD functionals with 6-311++G** and aug-cc-pvdz basis sets; together with those extracted from Imidazoleacetic acid hydrochloride crystal structure [12]. Apart from the C2–C3–N4 angle, all bond lengths and angles obtained for the two studied compounds, using all elected levels of theory, are comparable [13]. The C2–C3–N4 angle is *ca.* 5° larger for the I than for II tautomer, is in good agreement with a theoretical investigation of histamine tautomerism [14]. On the one hand, the hybrid functional (B3LYP) gave longer bond lengths compared to the LC-DFT functionals (CAM-B3LYP and ωB97XD); while the 6-311++G** basis set yielded longer bond lengths compared to those obtained from aug-cc-pvdz basis set. On the other hand, the bond angles are insensitive to the applied level of theory, in agreement with a study conducted by Wiberg [15].

**Table 1:** The bond lengths (Å) of the optimized geometries of H-bonded imidazole-4-acetic acid (I) and imidazole-5-acetic acid (II) using B3LYP, CAM-B3LYP and ωB97XD functionals with 6-311++G** (three top lines) and aug-cc-pvdz (three bottom lines) basis sets. The crystal structure of imidazole-4-acetic acid hydrochloride is given for comparison purposes.

Level of Theory	C1–N4		C1–N8		C2–N8		C2–C3		C3–N4		C3–C9	
	I	II	I	II	I	II	I	II	I	II	I	II
B3LYP	1.315	1.314	1.359	1.364	1.382	1.379	1.370	1.375	1.382	1.376	1.498	1.500
CAM-B3LYP	1.308	1.306	1.353	1.359	1.377	1.374	1.363	1.366	1.377	1.372	1.494	1.493
ωB97XD	1.310	1.308	1.353	1.359	1.377	1.374	1.365	1.368	1.377	1.372	1.495	1.493
B3LYP	1.319	1.312	1.361	1.360	1.383	1.375	1.376	1.372	1.384	1.374	1.500	1.494
CAM-B3LYP	1.313	1.312	1.354	1.360	1.379	1.375	1.369	1.372	1.379	1.374	1.497	1.494
ωB97XD	1.314	1.314	1.355	1.360	1.378	1.375	1.379	1.373	1.380	1.375	1.497	1.495
Expert ^a^	1.318		1.323		1.375		1.355		1.378		1.479	

^a^ Taken from [12].

**Table 2:** The bond angles (degrees) of the optimized geometries of H-bonded imidazole-4-acetic acid (I) and imidazole-5-acetic acid (II) using B3LYP, CAM-B3LYP, and ωB97XD functionals with 6-311++G** (three top lines) and aug-cc-pvdz (three bottom lines) basis sets. The crystal structure of imidazole-4-acetic acid hydrochloride is given for comparison purposes.

Level of Theory	N8–C1–N4		C1–N8–C2		N8–C2–C3		C3–N4–C1		C2–C3–N4	
	I	II	I	II	I	II	I	II	I	II
B3LYP	110.81	111.72	107.69	107.25	105.50	104.92	106.51	105.35	109.49	104.92
CAM-B3LYP	110.85	111.75	107.61	107.17	105.53	105.05	106.51	105.40	109.48	105.05
ωB97XD	110.99	111.88	107.63	107.17	105.44	105.02	106.29	105.22	109.64	105.02
B3LYP	110.78	111.69	107.81	107.42	105.46	104.88	106.49	105.29	109.46	104.88
CAM-B3LYP	110.84	111.72	107.74	107.34	105.49	105.01	106.48	105.34	109.45	105.01
ωB97XD	110.97	111.84	107.74	107.32	105.42	104.98	106.37	105.16	109.60	104.98
Expert ^a^	108.0		109.2		107.0		109.8		106.0	

^a^ Taken from [12].

Tables S3–S5 list the bond lengths, bond angles and dihedral angles of I and II computed by B3LYP/6-3111++G** level of theory. This is because this level of theory gives the most satisfactorily calculated geometrical parameters [15]. They were compared with those extracted from the crystal structure of Imidazole-acetic acid hydrochloride [12]. The absolute errors between the computed and measured values were also registered. The calculated bond lengths of the two substrates are comparable and, therefore, deviated almost equally from their crystal structure counterparts [12]. There is satisfactory agreement between the calculated and experimental bond lengths as indicated by the relatively small average error. The deviations between the calculated and measured bond angles and torsion angles are noticeable. These discrepancies could be explained by the different natures of the gas-phase and solid-phase geometries. The crystal structure intermolecular H-bonds could account, in part, for the large disparities between the calculated and experimental dihedral angles [16]. The largest percentage differences between the calculated and measured N8–C1 and C12–O13 bonds did not exceed *ca.* 3.1%; while the largest error encountered in C9–C12–O13 angle was of the order of *ca.* 3.5%.

**Figure 3:**
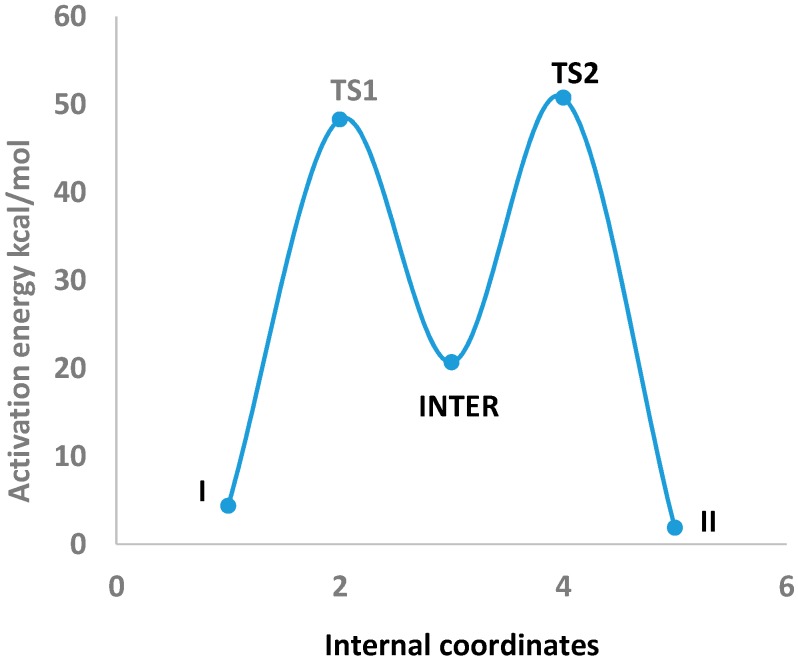
Intrinsic reaction coordinate (IRC) of the tautomerization of Imidazole-4-acetic acid (I) and Imidazole-5-acetic acid (II) through two transition states (TS1 and TS2) and an intermediate (INTER) which were obtained by using B3LYP/6-311++G** level of theory.

In Table 3 the optimized geometries of Imidazole-4-acetic acid (I) are listed that tautomerized to Imidazole-5-acetic acid (II) through two transition states (TS1 and TS2) and an sp^3^-type intermediate (INTER) which have been obtained by using B3LYP/6-311++G** level of theory (See Tables S6–S8). The tautomerization between I and II via these transitions states and the intermediate are depicted in Figure 3. They can be visualized through the following points: (1) The N4–H5 bond length of 1.008 Å in I elongated by 0.294 Å in TS1. It shortened and finally settled at 1.096 Å as a C3–H5 bond in the INTER. This is the first step of the 1,2-proton shift; (2) Consequently, the C3–N4 and C3–N8 bond lengths of I of 1.359 and 1.315 Å, expanded by 0.094 and 0.032 Å in TS1 and by another 0.022 and 0.108 Å in the INTER, respectively. These geometrical changes fit nicely with the proposed sp^3^-type intermediate formation [17]; (3) The INTER C3–H5 bond length of 1.096 Å elongated by 0.246 Å in TS2 and disappeared in II as a preparation for the second step of the 1,2-proton transfer; (4) The H5 proton then formed a partial bond with N8 of 1.310 Å, which finally settled as an N8–H5 bond (1.010 Å) in II. These remarks will further be supported by a natural bond orbital (NBO) analysis.

**Table 3:** Some selected optimized bond lengths of H-bonded imidazole-4-acetic acid (I) that tautomerized forming imidazole-5-acetic acid (II) through two transition states (TS1 and TS2) and an sp^3^-type intermediate (INTER) which were obtained by using B3LYP/6-311++G** level of theory.

Parameter	I	TS1	INTER	TS2	II
N4–H5	1.008	1.302	–	–	–
C3–N4	1.359	1.431	1.453	1.350	1.313
C3–H7	1.079	1.081	1.096	1.077	1.079
C3–N8	1.315	1.347	1.455	1.507	1.365
N8–C2	1.382	1.345	1.286	1.383	1.380
C3–H5	–	1.292	1.096	1.342	–
N8–H5	–	–	–	1.310	1.010

### 2.2. Thermodynamic Analysis

The values of Δ*E*, Δ*H*, Δ*S*, Δ*G*, and *K* for the tautomerization reaction: I↔II obtained by using B3LYP, CAM-B3LYP, and ωB97XD functionals with 6-311++G** and aug-cc-pvdz basis sets are shown in Table 4.

**Table 4:** B3LYP, CAM-B3LYP and ωB97XD functionals with 6-311++G** and aug-cc-pvdz basis sets zero-point reaction energies, enthalpies, entropies, free energies, and equilibrium constants for the equilibrium I↔II at 298.15 K.

Level of Theory	Δ*E*/kcal/mol	Δ*H*/kcal/mol	Δ*S*/cal/(mol K)	Δ*G*/kcal/mol	*K*
B3LYP/6-311++G**	−0.802	−0.952	−1.981	−0.361	1.84
CAM-B3LYP/6-311++G**	−0.750	−0.903	−1.905	−0.335	1.76
ωB97XD/6-311++G**	−0.863	−0.976	−1.406	−0.557	2.56
B3LYP/aug-cc-pvdz	−0.877	−1.039	−2.033	−0.433	2.08
CAM-B3LYP/aug-cc-pvdz	−0.841	−1.000	−2.127	−0.423	2.04
ωB97XD/aug-cc-pvdz	−0.838	−0.967	−1.589	−0.493	2.30

All elected levels of theory favor I over II by 0.750–0.877 kcal/mol, in excellent agreement with that obtained for the tautomers of nitro-imidazole of 0.760 kcal/mol using B3LYP/6-31G* level of theory [18]. Apparently, I is most favored when using ωB97XD/6-311++G** level of theory. The inclusion of the long-range correction on the traditional hybrid B3LYP has led to the CAM-B3LYP functional [19]. The effect of the long-range correction parameter on Δ*E* is seen in Table 4, in which B3LYP and CAM-B3LYP with 6-311++G** and aug-cc-pvdz basis sets results are compared. In these two cases, including the long-range correction changes Δ*E* by 0.052 and 0.036 kcal/mol, respectively. It brings about similar effects on Δ*G* of 0.026 and 0.010 kcal/mol, respectively; which implies respective decreases of *K* by 0.08 and 0.04. The implication of damped atom–atom dispersion corrections on long-range corrected hybrid density functionals yields ωB97XD functional [20]. The effect of dispersion correction on Δ*E*, Δ*G*, and *K* values are also listed in Table 4, in which the results of CAM-B3LYP and ωB97XD functionals with 6-311++G** and aug-cc-pvdz basis sets are compared. It is apparent that the dispersion correction lowers Δ*E* by 0.113 kcal/mol when using 6-311++G**, but it raises it by 0.003 kcal/mol when using aug-cc-pvdz basis set. In comparison, the dispersion-correction effect lowers ΔG when using both 6-311++G** and aug-cc-pvdz basis sets by 0.222 and 0.070 kcal/mol respectively; implying an increase of equilibrium constants at 298.15 K of 46% and 13%, respectively.

ΔG values at 298.15 K for all applied levels of theory are dictated mostly by ΔH (61%–67%) with moderately less contribution from TΔS (33%–39%). These percentage differences are attributed to long-range and dispersion corrections on functionals [15,16], rather than by basis set effects. The equilibrium constants values at 298.15 K range between 1.76 and 2.56. They are in reasonable agreement with those calculated for 4-nitro-imidazole↔5-nitro-imidazole equilibrium of 2.48 obtained from B3LYP/6-31+G* level of theory [13]. Their deviation from the experimental values of 0.45–1.5 [21], is attributable to phase difference *i.e.*, the experimental values are solution data, while ours are gas-phase estimates. Our calculated equilibrium values indicate that the equilibrium concentration of I is *ca.* twice that of II. The highest equilibrium concentration for I of more than 2.5-fold compared to that of II was obtained by using ωB97XD/6-311++G** level of theory. The negative values of ΔS indicate the preference of I over II at all temperatures.

### 2.3. Activation Energies

In Table 5 the zero-point electronic and activation energies of the tautomerization reaction are listed: I↔II. These parameters were computed by using B3LYP, CAM-B3LYP, and ωB97XD functionals with 6-311++G** and aug-cc-pvdz basis sets. The potential energy profile for this 1,2-proton shifts [17] tautomerization reaction applying B3LYP/6-311++G** level of theory is depicted in Figure 3. This level of theory has been selected because a remarkable agreement between the highly accurate Complete Basis Set (CBS) method and the B3LYP/6-311++G** level of theory was observed for computing the activation barriers of triazoles and tetrazoles [22]. The analysis of the normal modes of TS1 and TS2 imaginary frequencies (−1500.97 and −1533.54 cm^−1^, respectively) revealed the displacements of N4–H5 and C1–H6 bond lengths of I and INTER, in two 1,2-hydrogen shifts, to produce an sp^3^-type intermediate [17] and II. (See Table S9 in the Supplementary Information Section). The activation energies for this 1,2-proton shift tautomerization reaction obtained by all elected levels of theory are comparable, with a mean value of *ca.* 48.67 kcal/mol. These barrier heights are in good agreement when compared with an activation energy of 57.09 kcal/mol calculated for 1,2-proton shift for an H9 to H7-purine tautomerization using MP2/6-31+G** level of theory [17].

It is worth noting that these activation barriers are related to gas-phase conditions, and they are somewhat higher than the solution values obtained from placing 4-methylimidazole between ammonia and ammonium ion. The latter system is used as a model for triggering and facilitation of the proton relay mechanism for the H2-histamine receptor [23]. Our relatively high gas-phase 1,2-proton transfer activation energies mean that the interconversion between I and II could only occur under high thermal conditions.

**Table 5:** Zero-point electronic energies (a.u.) and activation energies (kcal/mol) of the tautomerization of imidazole-4-acetic acid (I) and imidazole-5-acetic acid (II) through two transition states (TS1 and TS2) and an intermediate (INTER) using B3LYP, CAM-B3LYP, and ωB97XD functionals with 6-311++G** and aug-cc-pvdz basis sets.

Level of Theory	Parameter	I	TS1	INTER	TS2	II
B3LYP/6-311++G**	Total energy	−454.132	−454.055	−454.106	−454.050	−454.131
	Act. energy	48.30	–	−31.99	–	50.80
CAM-B3LYP/6-311++G**	Total energy	−453.931	−453.852	−453.903	−453.846	−453.930
	Act. energy	49.55	–	−31.99	–	52.69
ωB97XD/6-311++G**	Total energy	−453.978	−453.901	−453.950	−453.895	−453.977
	Act. energy	48.30	–	−30.73	–	51.43
B3LYP/aug-cc-pvdz	Total energy	−454.065	−453.989	−454.039	−453.984	−454.063
	Act. energy	47.67	–	−31.36	–	49.55
CAM-B3LYP/aug-cc-pvdz	Total energy	−453.863	−453.785	−453.835	−453.779	−453.861
	Act. energy	49.92	–	−31.36	–	51.43
ωB97XD/aug-cc-pvdz	Total energy	−453.918	−453.841	−453.890	−453.836	−453.916
	Act. energy	48.30	–	30.73	–	50.18

Table 6 lists the gas-phase zero-point electronic and activation energies of the water-assisted 1,3-proton shift for the I↔II tautomerization reaction (See Figure S1 in the Supplementary Information Section) using the elected levels of theory. The barrier heights for this 1,3-hydrogen transfer range between 15.060–19.453kcal/mol. In Table 6 are, also, logged the zero-point electronic and activation energies of the 1,3-proton shift tautomerization using a first solvation layer of three water molecules. This environment has been simulated by using the PCM solvation model [24] with the CAM-B3LYP and ωB97XD functionals at 6-311++G** and aug-cc-pvdz basis sets. The barrier height decreased further to *ca.* 11.713 kcal/mol. These results indicate that the gas- or solution-phase I↔II tautomerization of imidazoleacetic acid are feasible at room temperature when assisted by water molecules.

**Table 6:** Zero-point electronic energies (a.u.) and forward (*E*a/f) and backward (*E*a/b) activation energies (kcal/mol) of the gas- and solution-phase water-assisted tautomerization of imidazole-4-acetic acid (I) and imidazole-5-acetic acid (II) through the transition state (TS) using the elected levels of theory.

Medium		Level of Theory	I	TS	II	*E*a/f	*E*a/b
Substrate I or II + 3H_2_O	gas	B3LYP/6-311++G**	−683.650	−683.619	−683.648	19.453	18.198
		CAM-B3LYP/6-311++G**	−683.369	−683.341	−683.368	17.570	16.943
		B3LYP/aug-cc-pvdz	−683.536	−683.505	−683.534	19.453	18.198
		CAM-B3LYP/aug-cc-pvdz	−683.255	−683.226	−683.253	18.198	16.943
		ωB97XD/6-311++G**	−683.318	−683.293	−683.317	15.876	15.047
		ωB97XD/aug-cc-pvdz	−683.421	−683.396	−683.420	15.938	14.997
	solution	CAM-B3LYP/6-311++G**	−683.382	−683.359	−683.389	14.433	18.825
		ωB97XD/6-311++G**	−683.337	−683.312	−683.331	15.835	12.093
		ωB97XD/aug-cc-pvdz	−683.441	−683.416	−683.435	15.685	11.713

On the one hand, when increasing the basis set from 6-311++G** to aug-cc-pvdz the barrier heights decreased by 0.63 kcal/mol for B3LYP, increased by 0.37 kcal/mol for CAM-B3LYP but remained constant for ωB97XD functional. On the other hand, the long-range correction elevated the barrier height by 1.25 and 2.25 kcal/mol when using 6-311++G** and aug-cc-pvdz basis sets, respectively. This means that the barrier height for imidazoleacetic acid tautomerization is both basis set and DFT functional dependent, but the effect is more conspicuous for the former.

### 2.4. Natural Bond Orbital (NBO) Analysis

**Figure 4:**
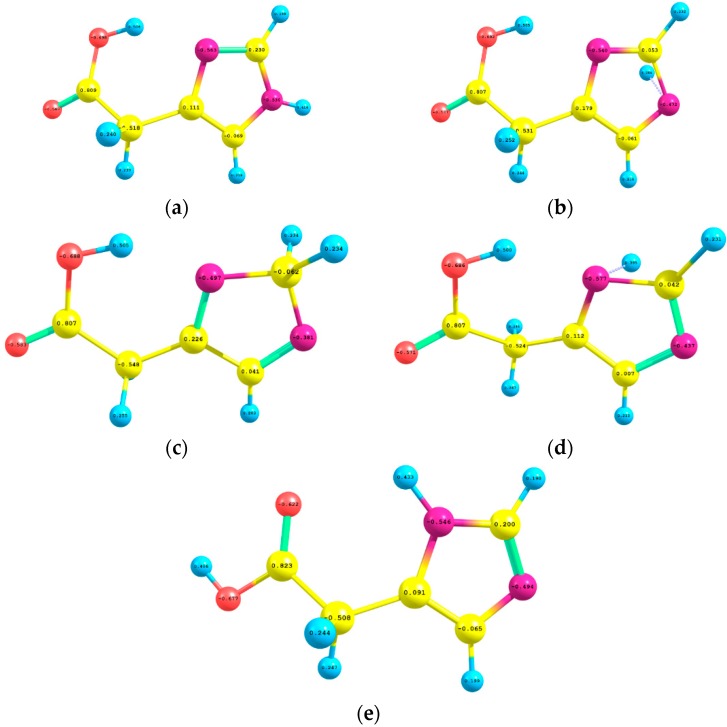
The natural atomic charges of (**a**) (I); (**b**) (TS1); (**c**) INTER; (**d**) TS2; and (**e**) II which were calculated using B3LYP/6-311++G** level of theory.

Figure 4 displays the natural atomic charges of I, TS1, INTER, TS2, and II which have been estimated by using B3LYP/6-311++G** level of theory. For I, the imidazole ring two nitrogen atoms N4 and N8 carried negative charges of −0.563e, −0.530e, respectively; while the carbon atom C1 and the future migrating hydrogen atom H6 acquired positive charges of 0.230e and 0.414e, respectively. In TS1, the natural atomic charges of the two nitrogen atoms (−0.540e, −0.472e) and the departing proton (0.384e) diminished a little bit; whilst that of C1 became almost neutral (0.053e). When H6 attached to C1 in the INTER, the natural atomic charges of N4, N8, C1, and H6 acquired the charges: −0.497e, −0.381e, −0.062e, and +0.234e, respectively. In TS2 and II, the natural atomic charges of the aforementioned atoms are almost similar to those of TS1 and I, respectively.

Table 7 transcribes the second-order perturbation (*E*_(2)_) computation of the hyperconjugative and hydrogen bonding energies of I, TS1, INTER, TS2, and II which have been calculated using B3LYP/6-311++G** level of theory. The application of Natural Bond Orbital (NBO) theory [25,26] facilitated the analysis of hydrogen bonding [27] and hyperconjugative [28] influences through using the second-order perturbation (*E*_(2)_) given by:

*E_(_*_2)_ = Δ*E*_ij_ = *q*_i_ (*F*_ij_)^2^/Δε (1)

where *q*_i_ denotes the occupancy of the donor orbital, F_ij_ estimates the off-diagonal NBO Kohn–Sham Matrix elements and Δε computes the difference between the energies of donor and acceptor orbitals i and j, respectively. Referring to Table 7, the most influential delocalizing interactions for I, TS1, INTER, TS2 and II are the N and O atoms lone-pairs as a result of the Lone-pair Effect [29]. On the one hand, the two nitrogen atoms lone pairs contributed totals of 106.67 and 92.74 kcal/mol for the stabilization of I and II, respectively. The strongest of which are the n_1N8_→π*_C1–N4_ (51.30kcal/mol for I and 46.06kcal/mol for II) and n_1N8_→π*_C2–C3_ (28.50 kcal/mol for I and 33.99 kcal/mol for II) interactions. On the other hand, those of the O atoms donated 102.76 and 100.42 kcal/mol to the stabilization of I and II, respectively; the most influential of which are the n_2O13_→π*_C12–O15_ (46.54 kcal/mol for I and 43.65 kcal/mol for II) and n_2O15_→σ*_C12–O13_ (30.65 kcal/mol for I and 32.22 kcal/mol for II) interactions. It is clear that the large difference between the N atoms lone pairs hyperconjugative energies of I and II of 13.93 kcal/mol, in favour of I, originates from the hydrogen bonding interaction n_1N4_→σ*_O13–H14_ that avails 15.07 kcal/mol for the stabilization of I. The other provenance of preference of I is attributed to the π*_C1–N4_→π*_C2–C3_ antibonding interaction that highly destabilized II by 84.56 kcal/mol compared to 46.64 kcal/mol for I.

There are some vicinal C1N4–C2C3* and C2C3–C1N4* π-interactions that contributed also to the stabilities of I and II [30]. The strong antiperiplanar [29] π_C1–N4_→π*_C2–C3_ donor-acceptor pair contributed 21.61 and 20.06 kcal/mol for I and II, respectively; while the less strong synperiplanar [28] π_C2–C3_→π*_C1–N4_ delocalization contributed by 14.95 and 14.80 kcal/mol for the stabilization of I and II, respectively.

**Table 7:** Second order perturbation (*E*_(2)_) estimation of the hyperconjugative energies (kcal/mol) of hydrogen bonded imidazole-4-acetic acid (I), the transition states (TS1 and TS2), the intermediate (INTER) and imidazole-5-acetic acid (II) which were calculated using B3LYP/6-311++G** level of theory.

Parameter	I	II	Parameter	INTER	Parameter	TS1	TS2
π_C1–N4_→π*_C2–C3_	21.61	20.06	σ_C1–N4_→σ*_C3–C9_	7.81	π_C1–N4_→π*_C2–C3_	13.24	11.84
π_C2–C3_→π*_C1–N4_	14.95	14.80	σ_C1–N8_→σ*_C2–H7_	6.12	π_C2–C3_→π*_C1–N4_	9.20	10.29
n_1N4_→σ*_C1–N8_	6.59	7.34	π_C2–N8_→π*_C3–N4_	14.42	n_1N4_→σ*_C1–N8_	4.57	5.01
n_1N4_→σ*_C2–C3_	5.21	4.85	π_C3–N4_→π*_C2–N8_	11.31	n_2N4_→π*_C1–N8_	23.01	17.54
n_1N4_→σ*_O13–H14_	15.07	˂0.5	σ_C9–H10_→π*_C3–N4_	5.15	n_2N4_→π*_C2–C3_	15.68	16.12
n_1N8_→π*_C1–N4_	51.30	46.06	σ_C9–H11_→π*_C3–N4_	5.15	n_1N8_→σ*_C1–N4_	3.84	4.47
n_1N8_→π*_C2–C3_	28.50	33.99	σ_O13–H14_→σ*_C12–O15_	5.48	n_1O13_→σ*_C9–C12_	3.01	2.89
n_2O13_→π*_C12–O15_	46.54	43.64	n_1N4_→σ*_C2–C3_	7.68	n_2O13_→π*_C12–O15_	23.16	22.32
n_2O13_→σ*_C9–C12_	5.92	˂0.5	n_1N4_→σ*_O13–H14_	18.96	n_2O15_→σ*_C9–C12_	10.07	9.98
n_2O15_→σ*_C9–C12_	19.65	17.55	n_1N8_→σ*_C1–N4_	5.07	n_2O15_→σ*_C12–O13_	15.33	15.76
n_2O15_→σ*_C12–O13_	30.65	32.22	n_1N8_→σ*_C2–C3_	7.39	σ_C1–N4_→n*_1H6_	31.64	15.22
π_C1–N4_→n*_1H6_	˂0.5	˂0.5	n_1O13_→σ*_C9–C12_	6.43	σ_C1–H5_→n*_1H6_	4.75	4.46
σ_C1–N8_→n*_1H6_	˂0.5	˂0.5	n_2O13_→π*_C12–O15_	50.42	π_C1–N8_→n*_1H6_	15.07	30.02
n_1N8_→n*_1H6_	˂0.5	˂0.5	n_2O15_→σ*_C9–C12_	20.20	n_1N8_→n*_1H6_	8.97	7.78
n_2N8_→n*_1H6_	˂0.5	˂0.5	n_2O15_→σ*_C12–O13_	30.19	n_2N8_→n*_1H6_	129.64	127.67
Total	247.99	223.51	Total	201.78	Total	311.18	301.28
π*_C1–N4_→π*_C2–C3_	46.64	84.56	–	–	n*_1H6_→π*_C1–N4_	730.01	347.08

The contribution of the aforementioned interactions to the stabilities of TS1 and TS2 diminished, paving the way for the ones between the imidazole ring atoms (N4, N8 and C1) and the migrating H6 atom. These delocalizations (π_C1–N4_→n*_1H6_, σ_C1–H5_→n*_1H6_, σ_C1–N8_→n*_1H6_, n_1N8_→n*_1H6_ and n_2N8_→n*_1H6_) yielded totals of 190.07 and 185.15 kcal/mol for their stabilities, respectively. These very high charge transfer interactions led to the debilitation of the N4–H6 and C1–H6 bonds and hence facilitated the transport of the proton from N4 to C1 and finally to N8 atom. These two 1,2-hydrogen shifts [17] produced an sp^3^-type intermediate (INTER) in the first step and II in the second jump.

Unlike I and II, the most influential hyperconjugative interactions in the INTER include σ→σ* charge transfers that contributed a total of 29.71 kcal/mol to its stability. The n→σ* or n→π* delocalizations contributed 146.34 kcal/mol for the stability of the INTER; compared to 209.43 and 186.65 kcal/mol for I and II, respectively. This could lead, safely, to the conclusion that the INTER is less stable than both I and II. The instabilities of TS1 and TS2 relative to I, II and INTER are estimated by 730.01 and 347.08 kcal/mol, respectively, due to the n*_1H6_→π*_C1–N4_ antibonding interaction.

All our elected levels of theory predicted relative stabilities of I and II in favor of the former by *ca.* 0.750–0.877 kcal/mol, which is below the chemical accuracy limit of 1.000 kcal/mol [13]. This fact necessitated the use of a more rigorous approach that applies the steric, electrostatic, or hyperconjugative interactions [30] for the assurance of these relative stabilities. The relative impacts of these three factors on the relative stabilities of I and II were resolved by using NBO computations applying the $DEL Keylist of the NBO version 3.1 [31] integrated within the Gaussian09 Suite [32]. The results comprising the total SCF, deletion and hyperconjugative energies of I and II using B3LYP/6-311++G** level of theory are shown in Table 8. The total SCF energy is indicative of the combined action of the three factors; where I is supported over II by 1.254 kcal/mol. The energy of deletion predicts the Lewis Structures of I and II where only the steric and electrostatic factors prevail. On the one hand, the Lewis Structures showed the preference of II over I by a huge amount of energy of 2068.542 kcal/mol as a result of minimal steric hindrance and smaller electrostatic repulsion in II compared to their effect in I. This is manifested by the crowdedness imposed by the closing up of the N4C3C9 angle in I (120.6°), compared to 123.9° in II (See Table S4) , which shortened the distance between N4 and H14 (1.832 Å) in I, compared to 2.255 Å between O15 and H6 in II. These situations brought about the formation of the hydrogen bonding in I. On the other hand, the delocalization interactions were competitive enough to avail 2069.796 kcal/mol for stabilization of I over II. That is, it exceeded the steric hindrance and the electrostatic repulsion energies of I by 1.254 kcal/mol. In the end, we can safely conclude that the preference of I over II is mainly due to hyperconjugation; or in particular to hydrogen bonding.

**Table 8:** NBO analyses of the total SCF, deletion and delocalization energies (a.u.) for hydrogen bonded imidazole-4-acetic acid (I) and imidazole-5-acetic acid (II) which were calculated by using B3LYP functional with 6-311++G** basis set.

Parameter	I	II	Δ*E* ^a^
Energy of Deletion (L)	−447.599	−450.897	+2068.542
Total SCF Energy (full)	−454.247	−454.245	−1.254
Delocalization Energy(NL)	−6.648	−3.348	−2069.796

^a^ Δ*E* = *E*_I_ − *E*_II_ kcal/mol.

### 2.5. Nonlinear Optical (NLO) Properties

In Table 9 are shown the nonlinear optical (NLO) properties of I and II which have been computed by applying B3LYP, CAM-B3LYP, and ωB97XD functionals with 6-311++G** and aug-cc-pvdz basis sets. We recorded the hyperpolarizabilities in atomic units (a.u.) which are equated to electrostatic units (esu) through the conversion factor: 1 a.u. = 8.6393 × 10^−33^ esu. The total hyperpolarizabilities (β_tot_) are given by the equation:

β_tot_ = [β_x_^2^ + β_y_^2^ + β_z_^2^]^½^ (2)

where

β_i_ = β_iii_ + ⅓∑(β_ijj_ + β_jij_ + β_jji_) (3)

In Table 9 are also listed the dipole moments (μ), the frontier orbitals (HOMOs and LUMOs) energies and the energy gaps (E.G.) of I and II, which were obtained by using the elected levels of theory. Some experimental [33] and theoretical [34] hyperpolarizability values for the prototypical NLO compound, p-nitro-aniline (pNA), are also given for the purpose of comparison. The dipole moments of I were predicted by all levels of theory to be more than double those of II. It is interesting to note that the inclusion of the long-range correction on the traditional hybrid functional [19] has increased the dipole moments by 2%–3% [35]; meanwhile the implication of the dispersion correction [20] has decreased them by almost the same amount. It is worth emphasizing that the changes of dipole moments are method rather than basis set dependent as the latter altered them by less than 1%. In addition, aug-cc-pvdz basis set yielded lower dipole moment values compared to 6-311++G**.

**Table 9:** Dipole moments (μ/Debye), Highest Occupied Molecular Orbital (HOMO) and Lowest Unoccupied Molecular Orbital (LUMO) energies (eV) and their energy gaps (E.G. = Δ*E*/eV), and total hyperpolarizability (β_tot_/a.u.) for I and II which were computed using B3LYP, CAM-B3LYP, and ωB97XD functionals with 6-311++G** and aug-cc-pvdz basis sets. The data for p-nitroaniline (pNA) is given for comparison purposes.

Level of Theory	Parameter	I	II	pNA ^a^
B3LYP/6311++G**	μ	8.59	3.92	7.16
	HOMO	−6.92	−6.40	–
	LUMO	−0.88	−0.92	–
	Δ*E*	6.04	5.48	4.29
	β_tot_	223	65	1327
CAM-B3LYP/6-311++G**	μ	8.79	4.04	7.23
	HOMO	−8.47	−7.88	–
	LUMO	−0.20	0.07	–
	Δ*E*	8.27	7.95	6.78
	β_tot_	143	19	1350
ωB97XD/6-311++G**	μ	8.59	4.04	7.16
	HOMO	−8.94	−8.40	–
	LUMO	0.58	0.87	–
	Δ*E*	9.52	9.27	7.96
	β_tot_	143	32	1350
B3LYP/aug-cc-pvdz	μ	8.52	3.88	–
	HOMO	−6.88	−6.35	–
	LUMO	−0.91	−0.93	–
	Δ*E*	5.97	5.42	–
	β_tot_	229	45	–
CAM-B3LYP/aug-cc-pvdz	μ	8.71	4.01	–
	HOMO	−8.42	−7.82	–
	LUMO	−0.26	0.00	–
	Δ*E*	8.16	7.82	–
	β_tot_	143	36	–
ωB97XD/aug-cc-pvdz	μ	8.54	3.99	–
	HOMO	−8.90	−8.34	–
	LUMO	0.45	0.74	–
	Δ*E*	9.35	9.08	–
	β_tot_	152	53	–
Expt ^b^	β_П_(−2ω;ω;ω)	–	–	1072 ± 44

^a^ Taken from [33]; ^b^ Taken from [34].

**Figure 5:**
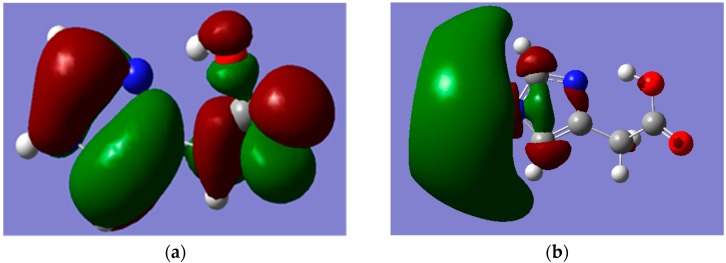
MOs of Hydrogen Bonded Imidazole-4-acetic acid (I) which were calculated by using B3LYP/6-311++G** level of theory. (**a**) HOMO; (**b**) LUMO. Note: carbon, oxygen, nitrogen and hydrogen atoms are in grey, red, blue and white colours, respectively.

**Figure 6:**
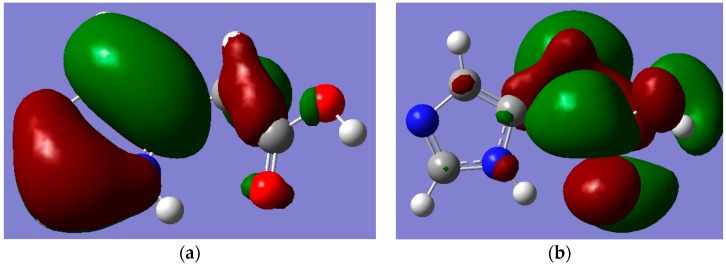
MOs of Hydrogen Bonded Imidazole-5-acetic acid (II) which were calculated by using B3LYP/6-311++G** level of theory. (**a**) HOMO; (**b**) LUMO. Note: carbon, oxygen, nitrogen and hydrogen atoms are in grey, red, blue and white colours, respectively.

**Figure 7:**
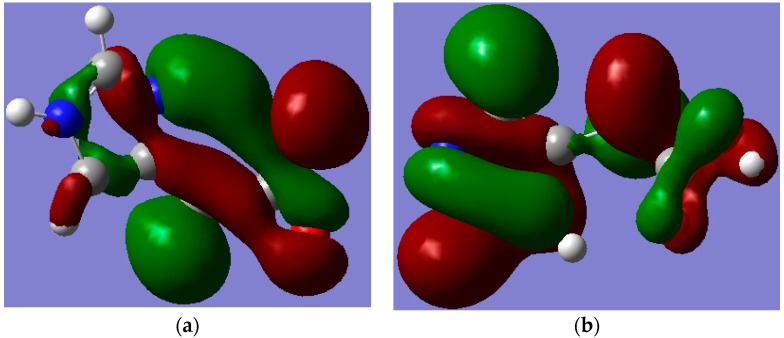
The pictorial visualization of HOMO-5 showing the Hydrogen bonding between the lone pair of the trigonal nitrogen atom of the imidazole ring in (**a**) (I) and its absence in (**b**) (II). Note: carbon, oxygen, nitrogen and hydrogen atoms are in grey, red, blue and white colours, respectively.

All tested levels of theory computed higher HOMO-LUMO energy gaps (E.G.) for I compared to II. Among the elected functionals, the traditional hybrid B3LYP method gave lower values compared to those estimated by the LC-DFT functionals (CAM-B3LYP and ωB97XD). That is, the effect of the long-range correction [19], has increased the E.G. by *ca.* 37% regardless of the basis set used; whilst the inclusion of dispersion correction [20] further widened them by *ca.* 15% independent of the applied basis set. Again, the basis set effect is minimal compared to that of the functionals. Figure 5 and Figure 6 depict the frontier molecular orbitals (FMOs) of I and II which have been pictured using B3LYP/6-311++G** level of theory. The HOMOs for both tautomers are delocalized mainly over the imidazole ring as π(C=N) and π(C=C) bonding orbitals, as well as oxygen atom lone pairs. The LUMO for I exits in the form of σ_p_(C–N and C–C) antibonding and nitrogen atom lone pair orbitals; while it occurs as π-antibonding orbitals over the acetate moiety for II. The strongly stabilizing H-bonding phenomenon that existed only for I is shown in Figure 7.

**Figure 8:**
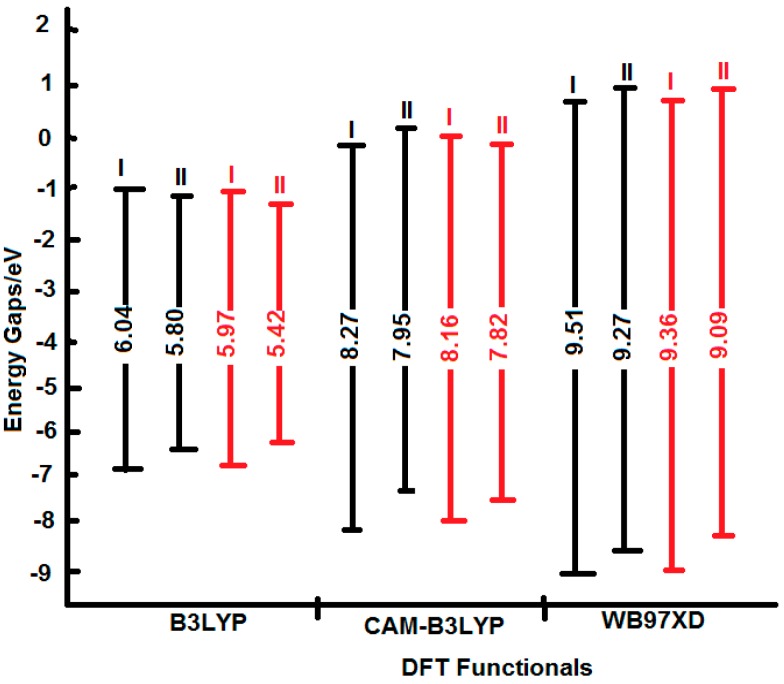
Schematic molecular orbital energy level diagrams of I and II tautomers which have been calculated using B3LYP, CAM-B3LYP, and xB97XD functionals with 6-311++G** (black lines) and aug-cc-pvdz (red lines) basis sets.

As displayed by Figure 8, the destabilization of the HOMOs and the stabilization of the LUMOs by the traditional hybrid functional (B3LYP) have led to lower E.G. The inclusion of the long-range and dispersion corrections (CAM-B3LYP and ωB97XD functionals) have progressively stabilized the HOMOs and destabilized the LUMOs. The net effect yielded higher E.G. that follows the sequence: ωB97XD > CAM-B3LYP > B3LYP. The 6-311++G** basis set computed a little bit higher E.G. in comparison to those obtained from aug-cc-pvdz. These findings are in excellent agreement with previous literature investigations [36,37,38].

The total hyperpolarizabilities (β_tot_) of the two tautomers estimated by using B3LYP, CAM-B3LYP, and ωB97XD functionals with 6-311++G** and aug-cc-pvdz basis sets are listed in Table 8. The β_tot_ values obtained from the traditional hybrid functional (B3LYP) are usually overestimated compared to those computed by LC-DFT functionals (CAM-B3LYP and ωB97XD) [39]. Apart from the value obtained for II using B3LYP/aug-cc-pvdz level of theory, this trend is met by the other values that can hardly show any dispersion correction effect [36]. This discrepancy could be due to the small β_tot_ values computed for II, which could probably encounter high percentage error. It is of interest to mention that the total hyperpolarizabilities of I are 3–5-fold higher than those of II. It is quite safe to conclude that the β_tot_ values are basis-set independent. The existence of H-bonding charge transfer in I could probably be responsible for the relatively enhanced values. This is exemplified by the n_N4_→σ*_O13–H14_ interaction that contributed 15.07 kcal/mol for the stabilization of I. However, compared to experimental [33] and theoretical [34] hyperpolarizabilities of pNA, all our calculated β_tot_ values are extremely small. As such, the imidazoleacetic acid molecule would not show any NLO properties. We judge this by: (1) the tetrahedral carbon atom that denied co-planarity between the imidazole ring and the acetate group; and (2) the absence of an electron-donating group for a perfect push-pull π-conjugated system [38]. Therefore, its NLO characters could probably be tailored and enormously enhanced by attaching an amino group, as an electron-donating entity, opposite to the electron-withdrawing acetate moiety.

Table 9 also lists the energy gaps (E.G.) for the two tautomers. A number of theoretical [40,41] and experimental [42] studies establish an inverse relation between the total hyperpolarizabilities and the HOMO-LUMO energy gaps. This criterion facilitates the eventuality of charge transfer that leads to higher hyperpolarizability. Our studied systems violate this relation. This is because the enhanced hyperpolarizabilities are dictated by many other factors. They include, in addition to small HOMO-LUMO energy gaps, planarity, large dipole moments, and presence of H-bonding and a push-pull mechanism. Both I and II are non-planar and devoid of a π-conjugated push-pull system. It is obvious that I has larger HOMO-LUMO energy gaps, compared to II; nonetheless, it gave relatively higher hyperpolarizabilities. This is, probably, because it has higher dipole moments as well as acquiring an H-bonding character.

## 3. Computational Details

The standard Gaussian09 programs [32] were used to carry out all calculations. The geometries of H-bonded imidazole-4-acetic acid (I) and imidazole-5-acetic acid (II) were fully optimized using density functional theory (DFT). The traditional hybrid exchange-correlation Becke, three-parameter, Lee–Yang–Parr (B3LYP [43,44]) the long-range corrected (LC-DFT) column-attenuating method (CAM-B3LYP [19]) and the LC-DFT with dispersion corrections, ωB97XD [20], functionals with the triple-zeta with polarization and diffuse functions on the hydrogen and heavy atoms basis set, 6-311++G** [45], and the augmented correlation-consistent polarized valence double-zeta, aug-cc-pvdz [46] basis sets were applied. The transition states (TS1 and TS2) of the interconversion between I and II was requested with the Berry Keyword [47] using B3LYP/6-311++G** level of theory. The Intrinsic Reaction Coordinate (IRC) [48] of the interconversion was monitored for the TSs that connected the minima in the potential energy profiles of I and II. The imaginary frequency designating the TSs and the IRC were investigated using GaussView [49] and Chemcraft [50] softwares.

The electric charges and hyperconjugative energies of I, TS1, INTER, TS2, and II were estimated using Version 3.1 of natural bond orbital (NBO) program [31] with the elected levels of theory. The idea here is to estimate the relative stabilities of the substrates using the donor-acceptor approach.

## 4. Conclusions

The gas-phase thermal tautomerization reaction between imidazole-4-acetic (I) and imidazole-5-acetic acid (II) was investigated theoretically and computationally using density functional theory (DFT). The traditional hybrid functional (B3LYP) and the long-range and dispersion corrected functionals (CAM-B3LYP and ωB97XD) were applied. The basis sets tested were 6-311++G** and aug-cc-pvdz. The geometrical parameters obtained for the two tautomers are comparable and agree satisfactorily with experimental ones [12]. The hybrid functional (B3LYP) estimated longer bond lengths compared to the LC-DFT functionals (CAM-B3LYP and ωB97XD); while the 6-311++G** basis set gave longer bond lengths compared to those obtained from aug-cc-pvdz basis set. All tested levels of theory favor I over II by 0.750–0.877 kcal/mol. The inclusion of the long-range and dispersion corrections affects Δ*E*, Δ*G*, and *K*. The gas-phase tautomerization reaction between I and II adopted a 1,2-proton shift mechanism, involving two transition states, with activation energies between 47.67 and 52.69 kcal/mol and an sp^3^-type intermediate. These data indicate that the interconversion between I and II, in the gas-phase, could only take place under severe thermal conditions.

Both I and II are characterized by high hyperconjugative interactions. The N and O atoms lone-pairs interactions with the imidazole ring and acetate group bonds contribute immensely to the stabilization of both tautomers. The origin of preference of I over II is attributed to the hydrogen bonding interaction: n_N4_→σ*_O13–H14_ that contributed 15.07 kcal/mol.

The total hyperpolarizability values of I are 3–5-fold greater than those for II despite the fact that the HOMO-LUMO energy gaps (E.G.) of the latter are smaller. This finding violates the inverse relation between total hyperpolarizability and E.G. values. This hurdle was overcome by the existence of H-bonding and higher dipole moments. The long-range correction is quite essential for total hyperpolarizability DFT evaluation as the traditional hybrid functional overestimate it. Finally, the computation of β_tot_ values is basis set independent.

## Supplementary Materials

Supplementary materials can be found at http://www.mdpi.com/1422-0067/16/11/25959/s1.
