# Supplementary material for: Gas-Phase Thermal Tautomerization of Imidazole-Acetic Acid: Theoretical and Computational Investigations
Source: Int J Mol Sci. 2015 Nov 4;16(11):26347–62. doi: 10.3390/ijms161125959 (PMC4661818; doi:10.3390/ijms161125959)
Supplement: Supplementary file 1 [file ijms-16-25959-s001.pdf]

International Journal of
Molecular Sciences

Supplementary Information

Table S1. Standard orientation of Imidazole-4-acetic acid (I) calculated by using B3LYP/6-311++G** level of theory.

Center Number	Atomic Number	Atomic Type	Coordinates (Angstroms)		
			X	Y	Z
1	6	0	2.156941	0.971888	−0.000035
2	6	0	1.612346	1.166228	−0.174279
3	6	0	0.527399	−0.430137	0.224228
4	7	0	0.886088	0.900511	0.328755
5	1	0	2.755146	1.868867	−0.033555
6	1	0	3.590335	−0.469487	−0.589396
7	1	0	1.741107	−2.217405	−0.368709
8	7	0	2.645807	−0.258257	−0.307819
9	6	0	−0.870198	−0.869754	0.536015
10	1	0	−1.029308	−0.845733	1.622119
11	1	0	−1.041969	−1.895647	0.212914
12	6	0	−1.995334	−0.009529	−0.062642
13	8	0	−1.762961	1.309337	−0.136162
14	1	0	−0.829076	1.505896	0.107560
15	8	0	−3.049093	−0.486801	−0.393489

Table S2. Standard orientation of Imidazole-5-acetic acid (II) calculated by using B3LYP/6-311++G** level of theory.

Center Number	Atomic Number	Atomic Type	Coordinates (Angstroms)		
			X	Y	Z
1	6	0	2.367548	0.875342	−0.063401
2	6	0	0.593872	−0.424682	0.161062
3	6	0	1.711494	−1.158283	−0.155295
4	7	0	2.809445	−0.339975	−0.291091
5	1	0	2.954950	1.780113	−0.100384
6	1	0	0.423086	1.679600	0.334120
7	7	0	1.033962	0.881488	0.229661
8	6	0	−0.807321	−0.850016	0.467709
9	1	0	−0.996497	−1.840264	0.052649
10	1	0	−0.963051	−0.938385	1.553420
11	6	0	−1.878486	0.110534	−0.005694
12	8	0	−3.028822	−0.526131	−0.305668
13	1	0	−3.682486	0.148763	−0.546921
14	8	0	−1.764616	1.312310	−0.069538
15	1	0	1.785007	−2.227222	−0.287510

Table S3. The bond lengths (Å) of the optimized geometries of H-bonded imidazole-4-acetic acid (I) and planar imidazole-5-acetic acid (II) using B3LYP/6-311++G** level of theory. The crystal structure of imidazole-4-acetic acid hydrochloride is given for comparison purposes.

Parameter	I	|Error|	II	|Error|	Crystal Structure [a]
C1-N4	1.315	0.003	1.314	0.004	1.318
N8-C1	1.359	0.036	1.364	0.041	1.323
N8-C2	1.382	0.007	1.379	0.004	1.375
C2-C3	1.370	0.015	1.375	0.020	1.355
N4-C3	1.382	0.004	1.375	0.003	1.378
C3-C9	1.498	0.019	1.500	0.021	1.479
C9-C12	1.538	0.021	1.511	0.004	1.507
C12-O13	1.341	0.029	1.350	0.037	1.313
C12-O15	1.203	0.009	1.209	0.003	1.212
Average		0.016	–	0.015	–

[a] Taken from [12].

Table S4. The bond angles (degrees) of the optimized geometries of H-bonded imidazole-4-acetic acid (I) and planar imidazole-5-acetic acid (II) using B3LYP/6-311++G** level of theory. The crystal structure of imidazole-4-acetic acid hydrochloride is given for comparison purposes.

Parameter	I	|Error|	II	|Error|	Crystal Structure [a]
C1-N4-C3	106.5	3.3	107.3	2.5	109.8
N4-C1-N8	110.8	2.8	111.7	3.7	108.0
C1-N8-C2	107.7	1.5	105.4	3.9	109.2
N8-C2-C3	105.5	1.5	110.7	3.7	107.0
C2-C3-N4	109.5	3.5	104.9	1.1	106.0
C2-C3-C9	129.9	0.7	131.1	0.5	130.6
N4-C3-C9	120.6	2.7	123.9	0.6	123.3
C3-C9-C12	115.9	1.9	114.7	0.7	114.0
C9-C12-O13	116.4	3.9	111.9	0.6	112.5
C9-C12-O15	121.7	1.4	125.5	2.4	123.1
O15-C12-O13	121.8	2.6	122.5	1.9	124.4
Average		2.35	–	1.96	–

[a] Taken from [12].

Table S5. The dihedral angles (degrees) of the optimized geometries of H-bonded imidazole-4-acetic acid (I) and planar imidazole-5-acetic acid (II) using B3LYP/6-311++G** level of theory. The crystal structure of imidazole-4-acetic acid hydrochloride is given for comparison purposes.

Parameter	I	|Error|	II	|Error|	Crystal Structure [a]
N4-C1-N8-C2	0.5	0.7	1.1	0.1	1.2
C1-N8-C2-C3	0.4	0.8	0.9	0.3	1.2
C3-C9-C12-O13	35	134.7	50.2	119.5	169.7
Average		45.4	–	39.97	–

[a] Taken from [12].

Table S6. Standard orientation of TS1 calculated by using B3LYP/6-311++G** level of theory.

Center Number	Atomic Number	Atomic Type	Coordinates (Angstroms)		
			X	Y	Z
1	6	0	2.184953	0.942481	−0.006118
2	6	0	1.660749	−1.165820	−0.154625
3	6	0	0.540006	−0.414060	0.218884
4	7	0	0.873386	0.889442	0.296642
5	1	0	2.828070	1.785681	0.205038
6	1	0	2.551765	0.537874	−1.177210
7	1	0	1.731642	−2.218484	−0.382566
8	7	0	2.738930	−0.354460	−0.248379
9	6	0	−0.857351	−0.880160	0.494083
10	1	0	−1.010315	−0.914712	1.580439
11	1	0	−1.014981	−1.891639	0.122126
12	6	0	−2.000372	−0.007380	−0.056241
13	8	0	−1.785289	1.314781	−0.092434
14	1	0	−0.850140	1.516721	0.135723
15	8	0	−3.050981	−0.491116	−0.382226

Table S7. Standard orientation of INTER calculated by using B3LYP/6-311++G** level of theory.

Center Number	Atomic Number	Atomic Type	Coordinates (Angstroms)		
			X	Y	Z
1	6	0	2.265686	0.966247	0.000047
2	6	0	1.804643	−1.160033	0.000003
3	6	0	0.537647	−0.383956	0.000026
4	7	0	0.813892	0.872453	0.000055
5	1	0	2.612497	1.515080	0.882566
6	1	0	2.612483	1.515137	−0.882442
7	1	0	1.894743	−2.241448	−0.000028
8	7	0	2.818505	−0.377280	−0.000003
9	6	0	−0.845238	−0.957600	0.000023
10	1	0	−0.966458	−1.614339	0.868309
11	1	0	−0.966426	−1.614428	−0.868198
12	6	0	−2.058583	−0.007263	−0.000043
13	8	0	−1.827055	1.307354	0.000008
14	1	0	−0.855943	1.486454	0.000052
15	8	0	−3.170771	−0.464484	−0.000128

Table S8. Standard orientation of TS2 calculated by using B3LYP/6-311++G** level of theory.

Center Number	Atomic Number	Atomic Type	Coordinates (Angstroms)		
			X	Y	Z
1	6	0	−2.334205	0.844292	−0.045128
2	6	0	−1.638307	−1.158156	−0.228985
3	6	0	−0.536115	−0.398346	0.180253
4	7	0	−0.922893	0.901339	0.261640
5	1	0	−2.876285	1.748837	−0.283230
6	1	0	−1.855224	1.037315	1.144974
7	1	0	−1.669146	−2.224003	−0.411217
8	7	0	−2.738010	−0.397770	−0.347190
9	6	0	0.851895	−0.830776	0.540993
10	1	0	1.015272	−1.867314	0.247866
11	1	0	0.985499	−0.788285	1.630140
12	6	0	2.011447	−0.011776	−0.051110
13	8	0	1.809544	1.310576	−0.196128
14	1	0	0.886723	1.548162	0.014331
15	8	0	3.066856	−0.516966	−0.318892

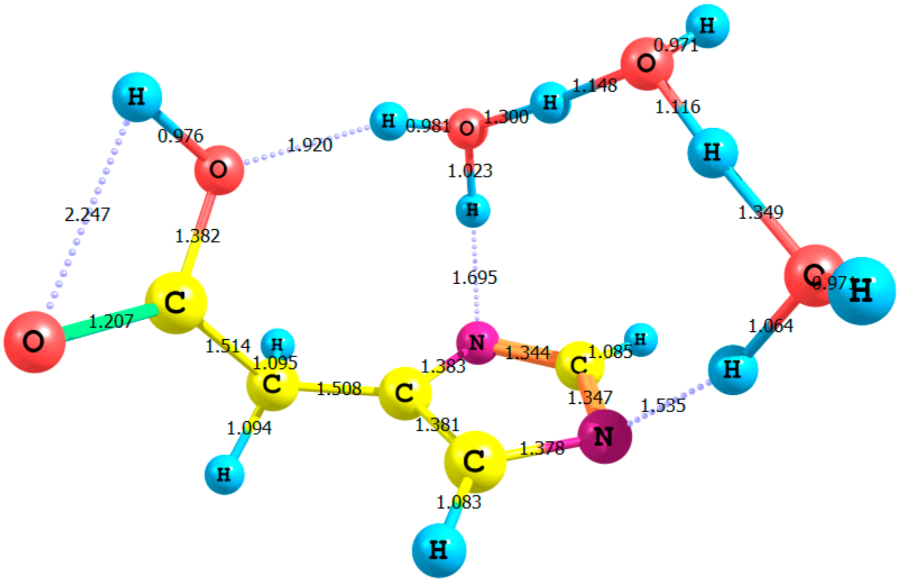

Figure S1. The structure of the Transition State for the 1,3-proton shift water-assisted tautomerization of I↔II.

Table S9. Imaginary frequencies (negative Signs) Harmonic frequencies (cm^{-1}), IR intensities (km/Mole), Reduced masses (amu), force constants (mDyne/Å), and normal coordinates calculated for TS1 (upper) and TS2 (lower) by using B3LYP/6-311++G** level of theory.

**Frequency**			−1500.97	
**Red. masses**			1.23	
**Frc consts**			1.64	
**IR Inten**			191.44	
**Atom**	**AN**	**X**	**Y**	**Z**
1	6	0.04	−0.01	−0.08
2	6	0.04	0.01	0.00
3	6	−0.01	0.03	0.01
4	7	−0.03	−0.02	0.00
5	1	0.02	−0.05	0.17
6	1	−0.44	0.87	0.03
7	1	0.00	0.00	−0.01
8	7	0.00	−0.07	0.04
9	6	0.00	0.00	0.00
10	1	0.01	0.00	0.00
11	1	0.00	0.00	0.00
12	6	0.00	0.00	0.00
13	8	0.00	0.00	0.00
14	1	0.00	0.00	0.00
15	8	0.00	0.00	0.00
**Frequency**			−1533.54	
**Red. masses**			1.24	
**Frc consts**			1.72	
**IR Inten**			121.13	
**Atom**	**AN**	**X**	**Y**	**Z**
1	6	−0.03	−0.03	−0.08
2	6	0.04	0.00	0.01
3	6	−0.01	−0.04	0.00
4	7	−0.06	0.03	0.04
5	1	−0.06	0.00	0.16
6	1	0.97	0.09	0.07
7	1	0.01	0.00	−0.01
8	7	−0.01	0.04	0.00
9	6	0.00	0.00	0.00
10	1	0.00	0.00	0.00
11	1	0.01	0.00	0.00
12	6	0.00	0.00	0.00
13	8	0.00	0.00	0.00
14	1	0.01	0.00	−0.01
15	8	0.00	0.00	0.00
